# Neglected ballistic trauma of the face in a young subject: A case report

**DOI:** 10.1016/j.amsu.2021.102852

**Published:** 2021-09-13

**Authors:** Mohamed Raiteb, Ulrich Opoko, Ayoub Sabr, Sanaa Elmrini, Amina Maadane, Faiçal Slimani

**Affiliations:** a Department of Stomatology and Maxillofacial Surgery, Hospital 20 Aout, CHU Ibn Rochd, B.P 2698, Casablanca, Morocco; b Faculty of Medicine and Pharmacy, Hassan II University of Casablanca, B.P 5696, Casablanca, Morocco

**Keywords:** Ballistic trauma, Firearm, Face, Management

## Abstract

Injuries by ballistic projectiles concern nowadays more and more frequently civilian populations. If the vital prognosis is rarely put at risk, the functional after-effects are frequent and important. The management of these injuries follows specific rules that must be known because they are sometimes different from the usual traumatology. However, it is important for any surgeon to understand the basic principles of ballistic injury. Indeed, the knowledge of the trajectory of the bullet and its final location allows to consider the potential injuries and to evaluate the management of the patient. CT is the examination of choice for penetrating foreign bodies, allowing for viewing of the entry site, bullet trajectory, possible scattered fragments, and, most importantly, a possible skull base breach, as well as providing useful information for planning the surgical procedure and, generally, for prognosis. The primary surgery must ensure an early and rigorous trimming associated with antibiotic therapy because the quality of the initial trimming significantly influences the final result, so this approach to the removal of the foreign body depends on its size, its anatomical location, the structures involved and the preference of the surgeon.

## Highlights

• Injuries caused by ballistic projectiles are nowadays more and more frequent in civilian populations.
• Any surgeon or emergency doctor may be confronted with such traumas, the management of which is governed by specific rules that must be known because they are sometimes different from usual traumatology.
• No physical theory can predict with certainty the behavior of a projectile in the human body.
• The knowledge of the trajectory of the bullet and its final location allows us to consider the potential injuries and to evaluate the management of the patient.

## 1 Introduction

Ballistic injuries are the consequence of the penetration into the body of a projectile or the contents of an explosive device. No physical theory can predict with certainty the behavior of a projectile in the human body [1,2]. Nowadays, projectile injuries are more and more frequent in civilian populations. Although the prognosis is rarely life-threatening, the functional after-effects are frequent and significant. The management of these injuries follows specific rules that must be known because they are sometimes different from the usual traumatology [3].

We report the case of a young man who suffered a ballistic facial trauma and discuss the diagnostic and therapeutic management.

This article has been reported in line with the PROCESS criteria [4].

## 2 Case report

**Fig. 1:**
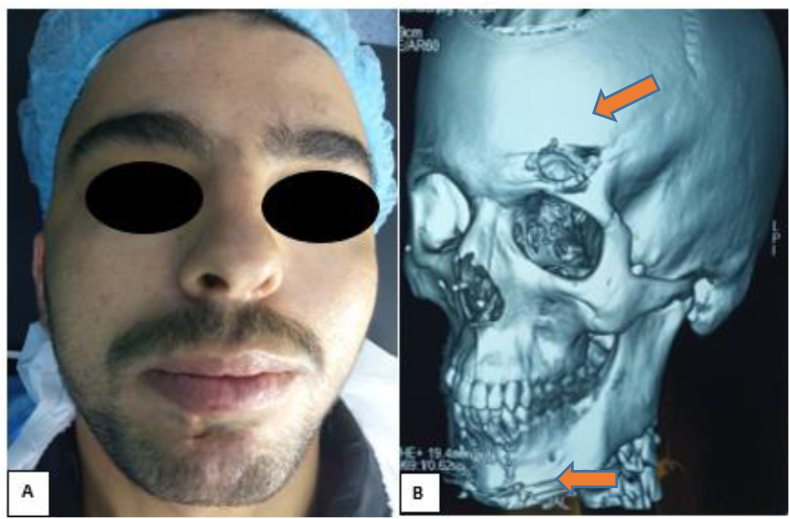
**A**: image of the patient showing scarred skin lesions of the projectile entry point at the left frontal and chin level, **B:** 3D reconstruction CT scan, showing foreign bodies of metallic appearance at the frontal and mandibular level.

**Fig. 2:**
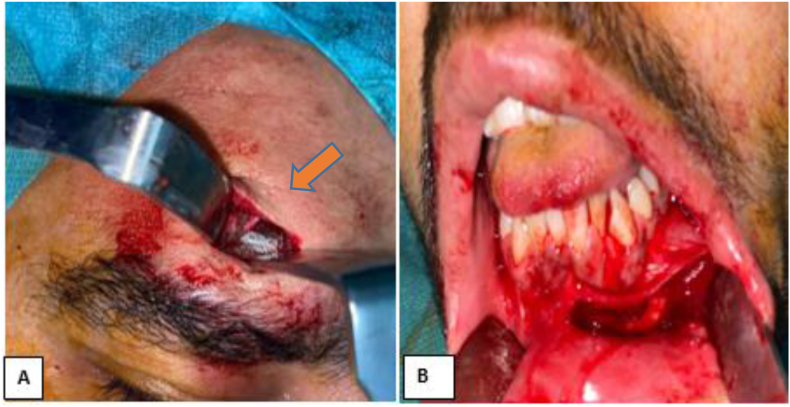
**A**: intraoperative image of the frontal surgical approach showing the ballistic projectile, **B**: intraoperative image of the lower vestibular endobuccal approach after extraction of the ballistic projectiles.

**Fig. 3:**
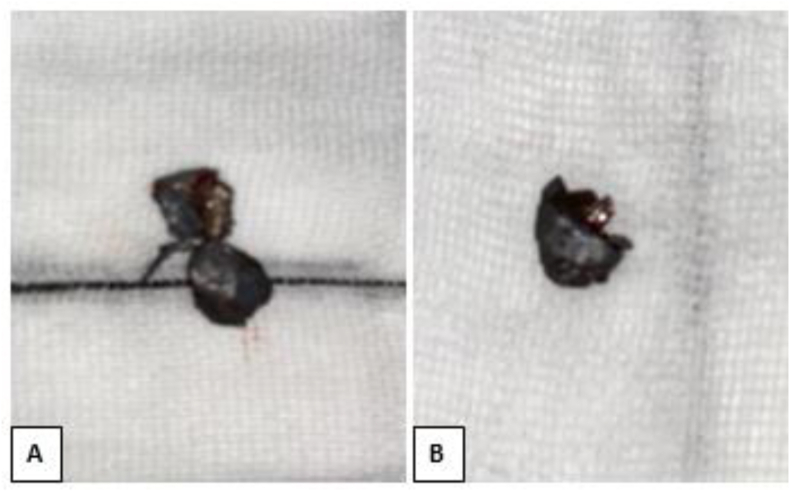
Images of metallic foreign bodies after extraction (A: frontal; B: mandibular).

A 25-year-old man, without any particular pathological history, victim of a firearm assault two years before his admission, during an attempted arrest, causing penetrating wounds of the face by projections of lead-based bullets.The patient had local care and extraction of some foreign bodies, but without an initial radiological workup including CT and/or X-ray. Two years later, the patient complained of pain in the chin and forehead area, which motivated him to consult our department. The clinical examination revealed scarring in the chin and left frontal area (Fig. 1A), sensitive to palpation, with perception of a mass in the subcutaneous area opposite the two scars, hard, mobile in the deep plane, attached to the remaining metal projectiles. A CT scan was requested this time, which confirmed the presence of metallic foreign bodies in the soft tissues coming into contact with the bone, without any bony continuity, at the right frontal level and at the symphyseal level (Fig. 1B). However, the patient had no clinical signs of lead poisoning. After informed and written consent from the parents, he underwent extraction of these metallic foreign bodies under general anesthesia (Fig. 2). Intraoperatively, they were really ballistic projectiles (Fig. 3). We performed a frontal approach using the old frontal scar, and a lower vestibular endobuccal approach (Fig. 2).

The postoperative period was favourable, without complications, with medical treatment based on antibiotics (amoxicillin + clavulanic acid: 80mg/kg/day in 3 doses) and analgesics (paracetamol: 15mg/kg/day every 6 hours). Follow-up at the post-discharge consultation was done at 15 days, then 1,3 and 6 months later, without any complications being objectified.

## 3 Discussion

Ballistic trauma is not limited to the battlefields and is currently spreading more and more to civilian environments. Any surgeon or emergency physician may be confronted with such injuries, the management of which is specific [5].

The incidence of craniofacial ballistic trauma is quite difficult to evaluate because of the frequency of death of victims at the scene: almost 50% of them die instantly. In survivors, the pathophysiological consequences depend on the kinetic energy. A high-energy projectile causes a permanent cavity with ischemic and hemorrhagic phenomena at its periphery [6].

On the other hand, no physical theory can predict with certainty the behavior of a projectile in the human body. However, it is important for any surgeon to understand the basic principles of ballistic injury. Indeed, the knowledge of the trajectory of the bullet and its final location allows to consider the potential lesions and to evaluate the management of the patient.

The first particularity of the case we report lies in the fact that the trajectory of our projectiles was simple to determine, with the clinical examination revealing entry doors characterized by scarring lesions on the forehead and chin, and the absence of exit doors, but nevertheless these projectiles were in contact with the bones (frontal and mandible) without causing a solution of bony continuity. This could be related to the great distance of the shot, to the trajectory of the bullet with a low relative speed of the projectile at the time of the contact with the patient. Indeed, the speed of the projectile decreases with distance due to the resistance of the air to its progression [1,2,7].

The second feature is the absence of life-threatening injuries during the migration of the bullet in this patient. Firearm injuries vary depending on the nature of the projectile, muzzle velocity, distance and body part involved [7].

Craniofacial CT with three-dimensional reconstruction is the examination of choice for penetrating foreign bodies, allowing to see the site of entry, the trajectory of the bullet, possible scattered fragments and, most importantly, a possible skull base breach, as well as to provide useful information for planning the surgical procedure and, generally, for the prognosis. However, this examination is still limited by metal artifacts from the bullet and patient motion artifacts.

MRI can provide valuable additional data for non-metallic objects, especially if intracranial lesions or osteomeningeal breach are suspected [6,8].

Normally, treatment should be undertaken as an emergency to avoid the risk of complications, especially infectious ones. Primary surgery must ensure early and rigorous trimming associated with antibiotic therapy because the quality of the initial trimming has a significant influence on the final result and the extraction of the bullet is performed first [3,6].

The approach to removal of the foreign body depends on its size, anatomical location, structures involved and surgeon preference [8].

However, in our case, the management was incomplete at the outset of his ballistic trauma, marked by the absence of an initial CT scan to assess the patient's injury status and to reassure oneself of the presence or absence of remaining metal projectiles at the time of initial care.

Bedry R et al. reported in their study that even with a large number of bullets in the body, the blood lead level increases to low values without causing any clinical sign, except in exceptional circumstances [8]. Thus, in our case, given that our patient did not present any clinical symptoms of lead poisoning despite the fact that the trauma was two years old, we did not find it necessary to perform a blood lead level test and no chelation was performed.

The projectiles were later successfully removed by the frontal and inferior vestibular *trans*-lesion approach, which was chosen because of its considerably lower morbidity and less aesthetic damage [9].

## 4 Conclusion

Projectile wounds group together pluritissular and heterogeneous lesions. This case of facial ballistic trauma shows the importance of imaging in the management of the patient. An adequate management, with a complete initial lesion assessment would lead to an early management. The treatment of these projectile injuries must always remain adapted to the wound and not to the weapon; and the surgical approach must be done by specialist surgeons mastering the anatomy.

## Patients concent

Written informed consent was obtained from the parents of the minor girl and the second patient for the publication of this case report and accompanying images. A copy of the written consents is available upon request for review by the editor of this journal.

## Sources of funding

None.

## Provenance and peer review

Not comissioned, externaly peer reviewed.

## Autors contribution

- Corresponding author, and writing the paper: Ulrich Opoko;

- Writing the paper: Mohamed Raiteb, Ayoub Sabr, Sanaa Elmrini, Amina Maadane;

- Correction of the paper: Faiçal Slimani.

## Ethical approval

Our study is exempted of ethical approval.

## Registration of research studies

1. Name of the registry:

2. Unique Identifying number or registration ID:

3. Hyperlink to your specific registration (must be publicly accessible and will be checked):

## Guarantor

OPOKO ULRICH.

## Declaration of competing interest

Authors of this article have no conflict or computing interest.
